# Supplementary material for: Glucocorticoids unleash immune-dependent melanoma control through inhibition of the GARP/TGF-β axis
Source: Cancer Discov. Author manuscript; Available in PMC 2025 Oct 23. (PMC7618275; doi:10.1158/2159-8290.CD-24-1224)
Supplement: 7 [file EMS209516-supplement-7.pdf]

Figure S1

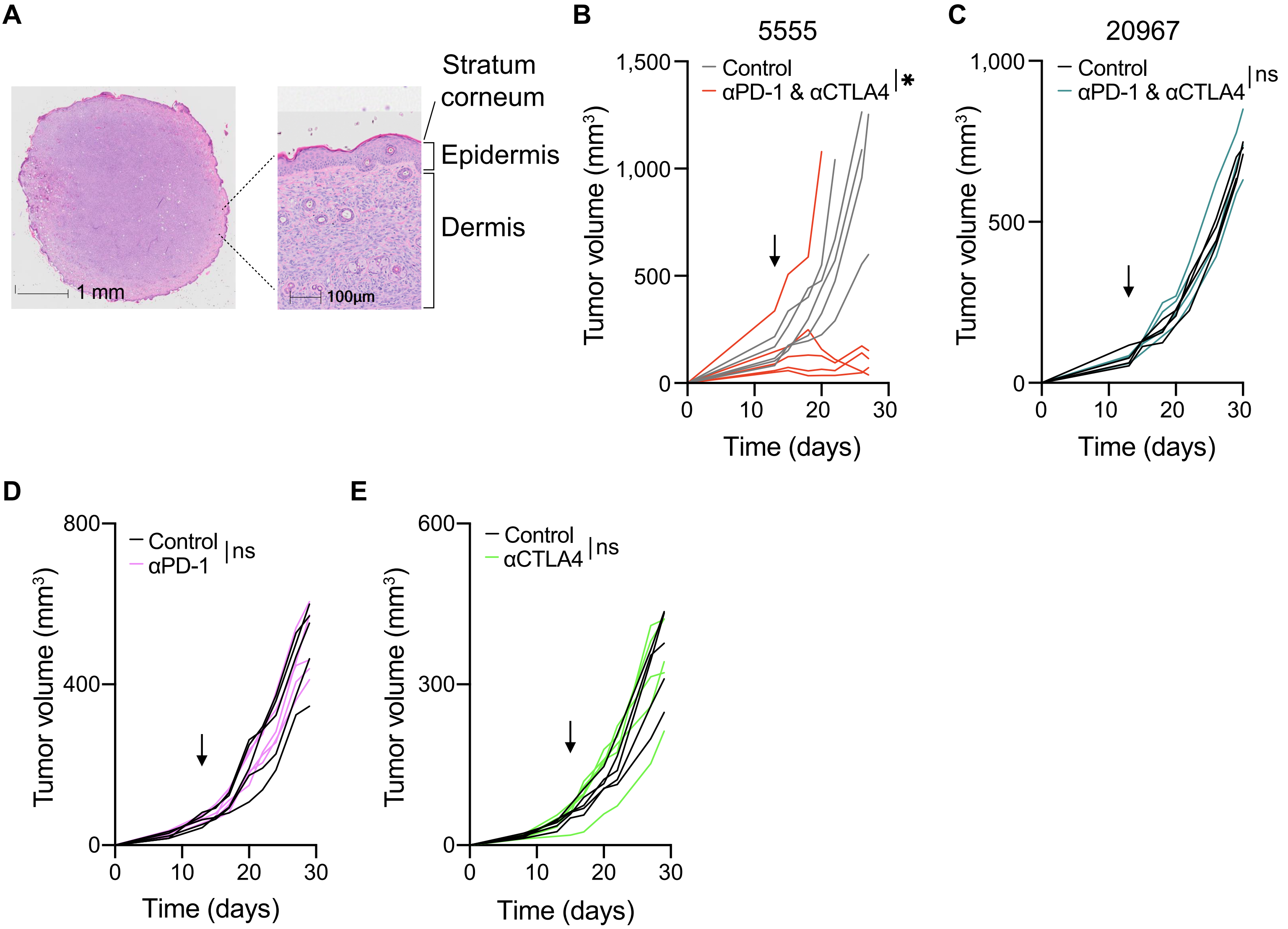

A
Stratum corneum
Epidermis
Dermis
1 mm
100µm
B
5555
Control
αPD-1 & αCTLA4
*
Tumor volume (mm³)
Time (days)
C
20967
Control
αPD-1 & αCTLA4
ns
Tumor volume (mm³)
Time (days)
D
Control
αPD-1
ns
Tumor volume (mm³)
Time (days)
E
Control
αCTLA4
ns
Tumor volume (mm³)
Time (days)

Supplementary Figure 1. The 20967 melanoma model is resistant to immune checkpoint blockade.

(**A**) Representative hematoxylin and eosin slide of an intradermal 20967 melanoma tumor.

(**B-E**) Individual tumor growth profiles of 5555 (B) and 20967 tumors (C-E) after treatment with combination αPD-1 and αCTLA4 (B, C), αPD-1 only (D) or αCTLA4 only (E) (n=5 per group). Arrow indicates start of treatment.

Two-way ANOVA (B-E). *, $P < 0.05$; ns, not significant.
