# Supplementary material for: Glucocorticoids unleash immune-dependent melanoma control through inhibition of the GARP/TGF-β axis
Source: Cancer Discov. Author manuscript; Available in PMC 2025 Oct 23. (PMC7618275; doi:10.1158/2159-8290.CD-24-1224)
Supplement: 8 [file EMS209516-supplement-8.pdf]

Figure S2

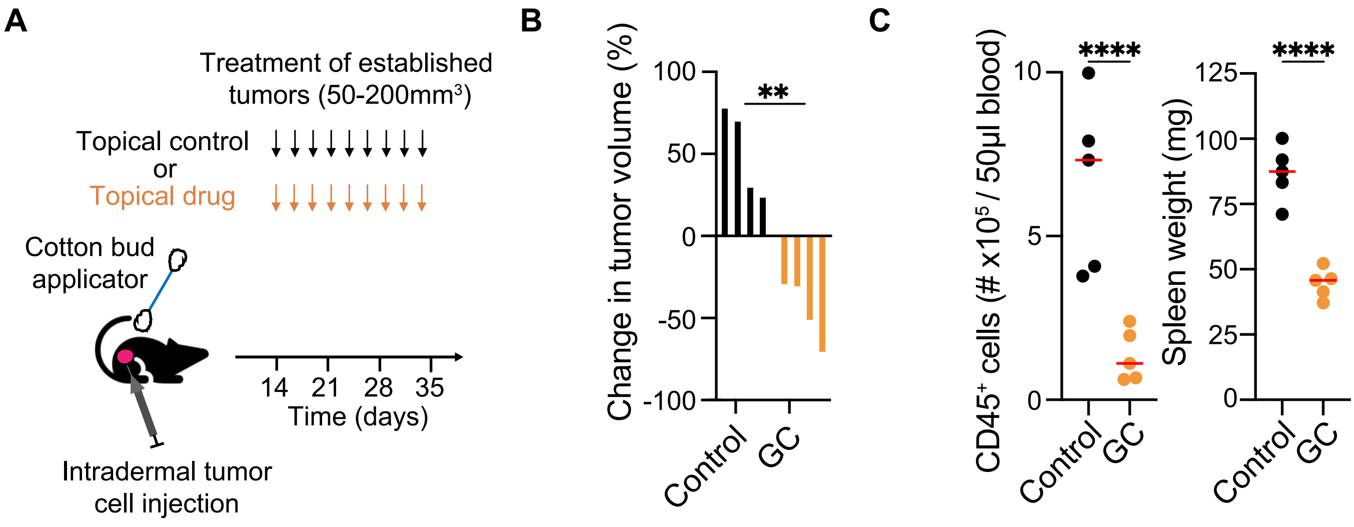
A
Treatment of established tumors (50-200mm³)
Topical control
or
Topical drug
Cotton bud applicator
Intradermal tumor cell injection
14 21 28 35
Time (days)
B
Change in tumor volume (%)
100
50
0
-50
-100
**
Control
GC
C
CD45⁺ cells (# x10⁵ / 50µl blood)
10
5
0
****
Control
GC
Spleen weight (mg)
125
100
75
50
25
0
****
Control
GC

Supplementary Figure 2. Topical GC treatment of 20967 tumors reduce leukocyte counts systemically.

(**A**) Treatment schedule of melanoma with topical drug.

(**B**) Waterfall plot showing percentage change in tumor volume at day 5 post-treatment of 20967 tumors over $300mm^3$ following control and GC-treatment (n=4 per group).

(**C**) Peripheral blood $CD45^+$ cell count on day 14 after treatment start (left panel) and spleen weight (right panel) on day 5 post treatment start with GC or control (n=5 per group).

Data are expressed as mean; unpaired t-test (B, C). **, $P < 0.01$; ****, $P < 0.0001$.
