# Supplementary material for: Glucocorticoids unleash immune-dependent melanoma control through inhibition of the GARP/TGF-β axis
Source: Cancer Discov. Author manuscript; Available in PMC 2025 Oct 23. (PMC7618275; doi:10.1158/2159-8290.CD-24-1224)
Supplement: 9 [file EMS209516-supplement-9.pdf]

Figure S3

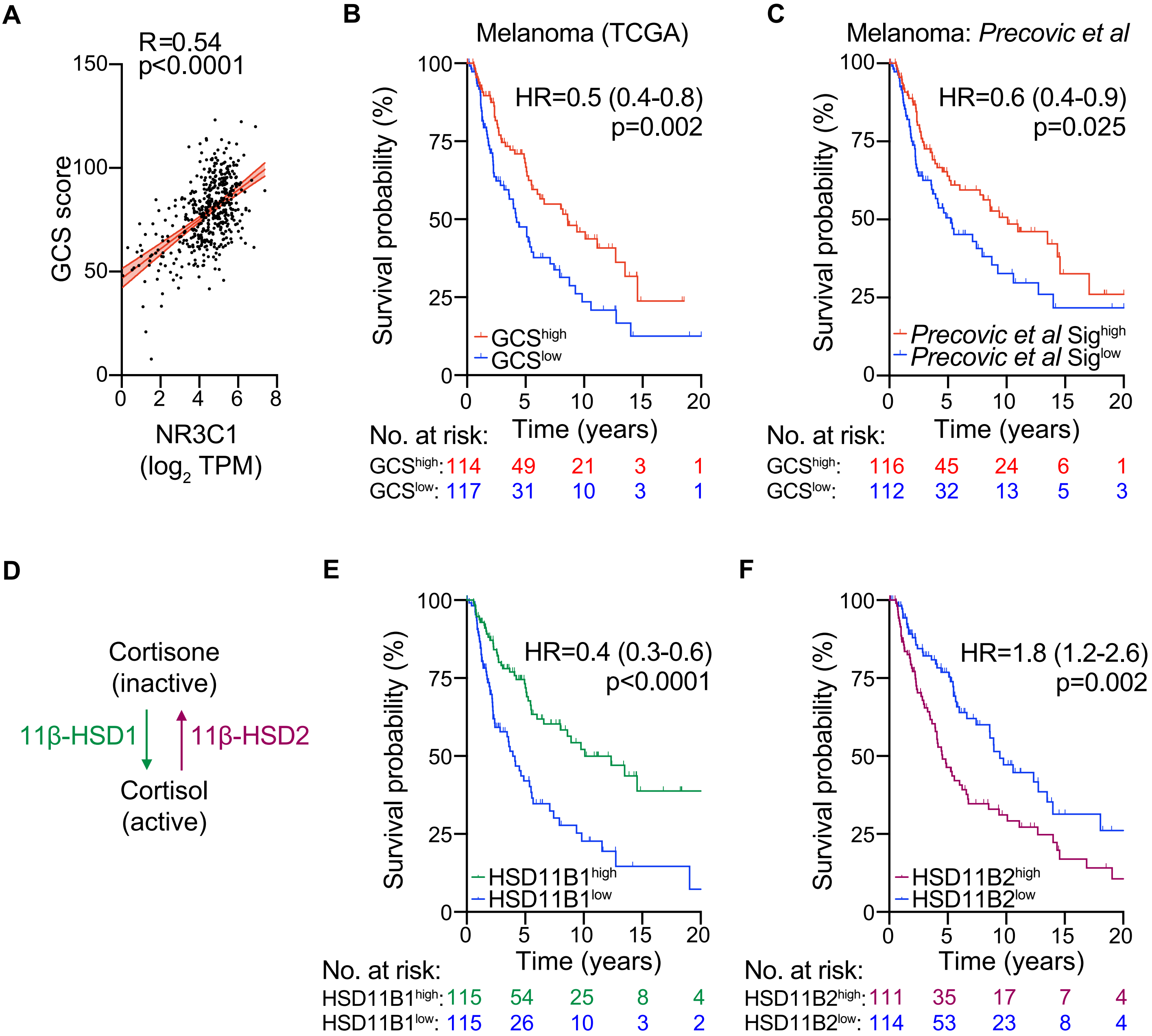
A
R=0.54
p<0.0001
GCS score
NR3C1
(log2 TPM)
B
Melanoma (TCGA)
HR=0.5 (0.4-0.8)
p=0.002
Survival probability (%)
GCShigh
GCSlow
Time (years)
No. at risk:
GCShigh: 114 49 21 3 1
GCSlow: 117 31 10 3 1
C
Melanoma: Precovic et al
HR=0.6 (0.4-0.9)
p=0.025
Survival probability (%)
Precovic et al Sighigh
Precovic et al Siglow
Time (years)
No. at risk:
GCShigh: 116 45 24 6 1
GCSlow: 112 32 13 5 3
D
Cortisone
(inactive)
11β-HSD1
11β-HSD2
Cortisol
(active)
E
HR=0.4 (0.3-0.6)
p<0.0001
Survival probability (%)
HSD11B1high
HSD11B1low
Time (years)
No. at risk:
HSD11B1high: 115 54 25 8 4
HSD11B1low: 115 26 10 3 2
F
HR=1.8 (1.2-2.6)
p=0.002
Survival probability (%)
HSD11B2high
HSD11B2low
Time (years)
No. at risk:
HSD11B2high: 111 35 17 7 4
HSD11B2low: 114 53 23 8 4

Supplementary Figure 3. Intratumoral GC receptor expression, signaling and levels are associated with improved survival in melanoma.

(**A**) Correlation, with 95% confidence interval shown, between GR expression and GC Signature in melanoma patients (TCGA, n=458).

(**B, C**) KM survival analysis based on GC signature (GCS; described later, Table S2, B) or previously published GC signature (C), stratified by upper and lower quartiles, of TCGA melanoma (n=458) patients.

(**D**) Pathway controlling active glucocorticoid levels in peripheral tissues.

(**E, F**) KM survival plots of TCGA melanoma patients (n=458) stratified by upper and lower quartile of 11β-HSD1 (F) and 11β-HSD2 expression (G).

Linear regression with Pearson correlation (A), Hazard ratio (95% confidence interval) or log-rank (Mantel-Cox) test (B, C, E, F).
