# Supplementary material for: Glucocorticoids unleash immune-dependent melanoma control through inhibition of the GARP/TGF-β axis
Source: Cancer Discov. Author manuscript; Available in PMC 2025 Oct 23. (PMC7618275; doi:10.1158/2159-8290.CD-24-1224)
Supplement: 10 [file EMS209516-supplement-10.pdf]

Figure S4

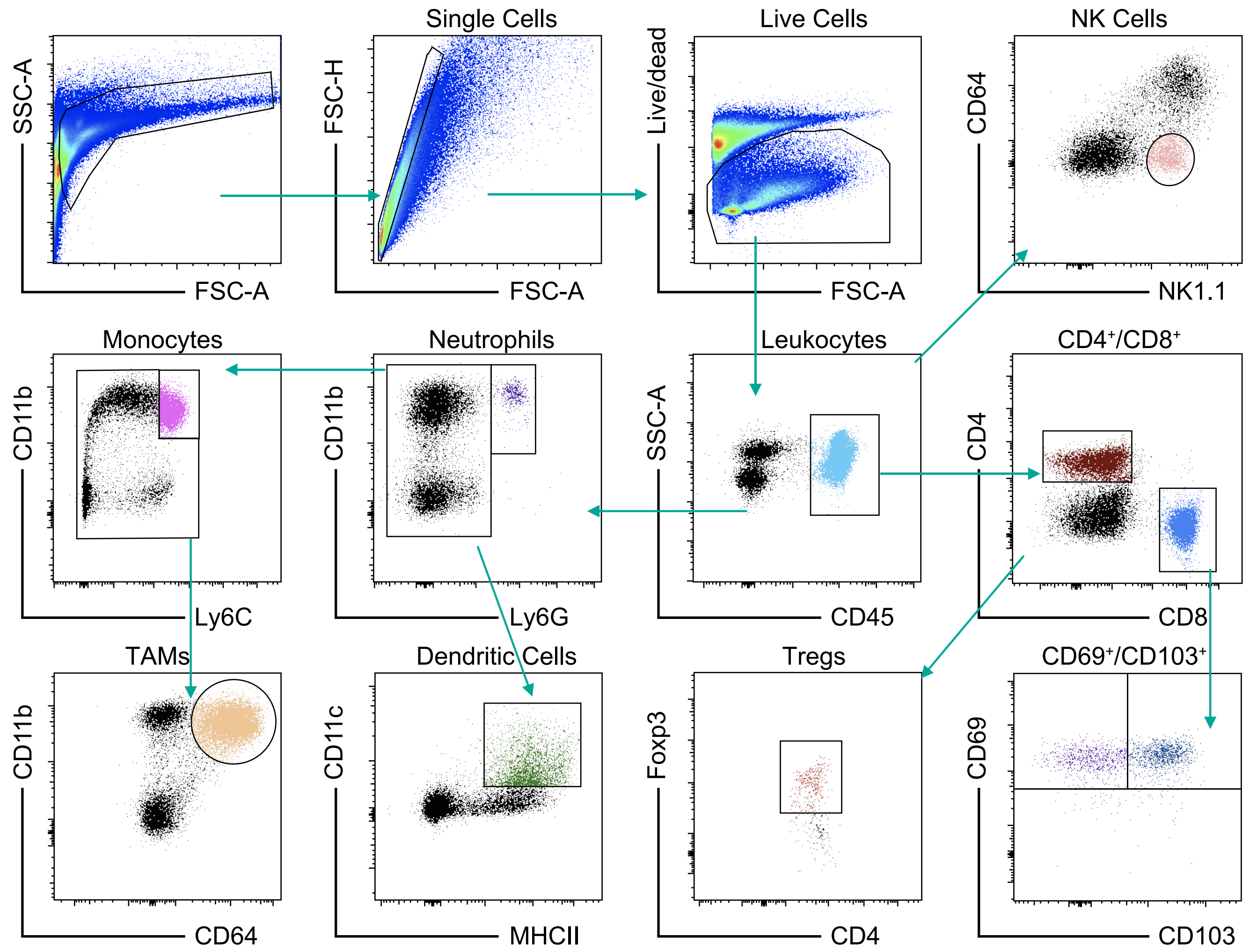

SSC-A
FSC-A
Single Cells
FSC-H
FSC-A
Live Cells
Live/dead
FSC-A
NK Cells
CD64
NK1.1
Monocytes
CD11b
Ly6C
Neutrophils
CD11b
Ly6G
Leukocytes
SSC-A
CD45
CD4+/CD8+
CD4
CD8
TAMs
CD11b
CD64
Dendritic Cells
CD11c
MHCII
Tregs
Foxp3
CD4
CD69+/CD103+
CD69
CD103

Supplementary Figure 4. Gating strategy used for intratumoral immune infiltrate analysis.

Gating strategy for intratumoral immune-infiltrate analysis of 20967 melanomas (related to Figures 2, 3 and Supplementary Figure 5).
