# Supplementary material for: Glucocorticoids unleash immune-dependent melanoma control through inhibition of the GARP/TGF-β axis
Source: Cancer Discov. Author manuscript; Available in PMC 2025 Oct 23. (PMC7618275; doi:10.1158/2159-8290.CD-24-1224)
Supplement: 11 [file EMS209516-supplement-11.pdf]

Figure S5

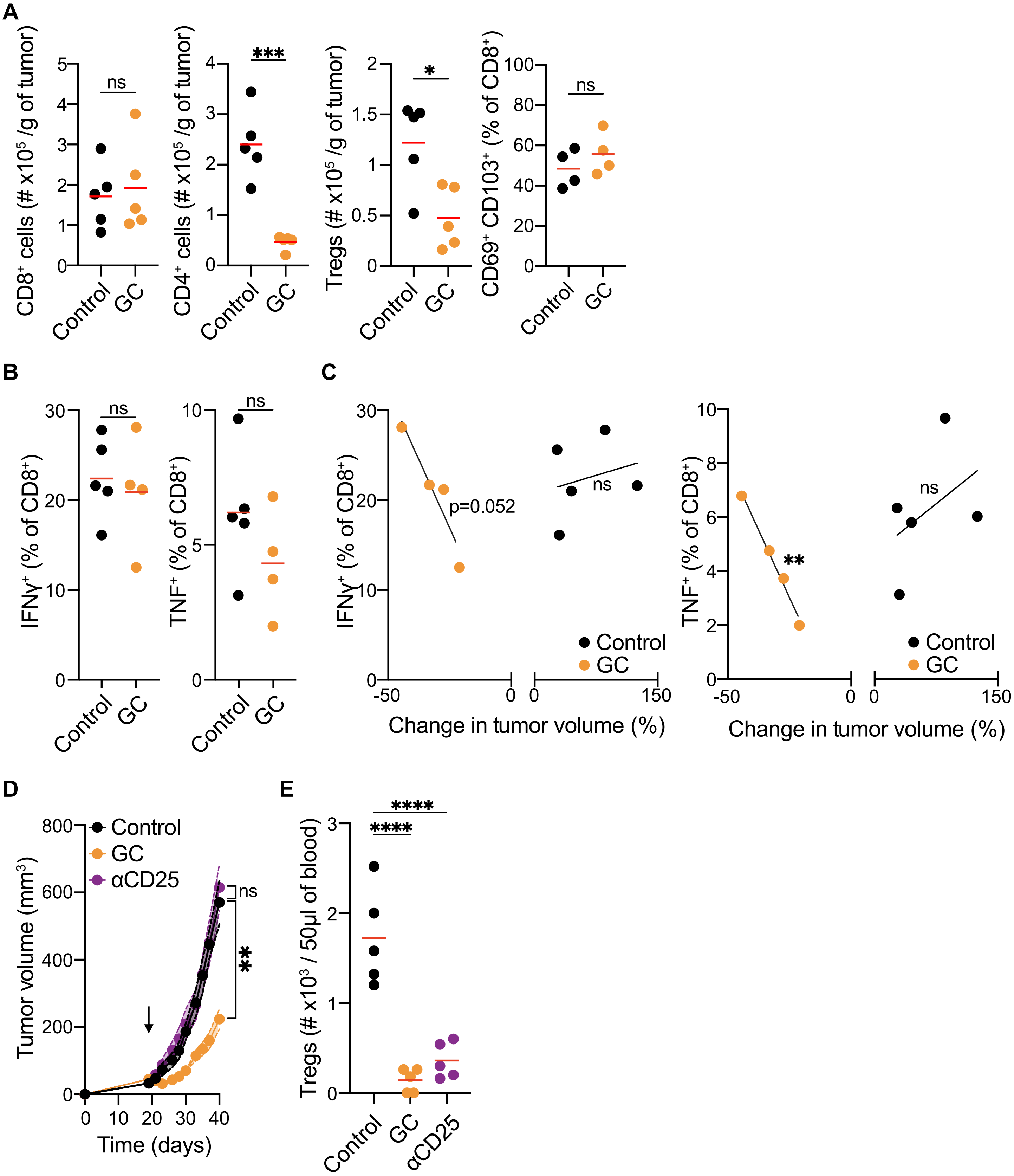
A
CD8+ cells (# x10^5 /g of tumor)
CD4+ cells (# x10^5 /g of tumor)
Tregs (# x10^5 /g of tumor)
CD69+ CD103+ (% of CD8+)
ns
***
*
ns
Control
GC
B
IFNγ+ (% of CD8+)
TNF+ (% of CD8+)
ns
ns
C
p=0.052
ns
**
Control
GC
Change in tumor volume (%)
D
Tumor volume (mm3)
Time (days)
Control
GC
αCD25
ns
**
E
Tregs (# x10^3 / 50µl of blood)
****
****
Control
GC
αCD25

Supplementary Figure 5. CD8$^+$ T cell activity correlates with extent of GC-induced tumor shrinkage, and Treg depletion does not phenocopy GC treatment.

(**A**) Intratumoral immune-infiltrate analysis of 20967 melanomas on day 5 post-treatment with control or GCs (n=5 per group).

(**B, C**) Intracellular cytokine production analysis of CD8$^+$ T cells in control or GC-treated melanomas on day 5 post-treatment (n=4-5 per group) showing proportion (B) or correlation with change in tumor volume (C).

(**D, E**) 20967 tumor growth curves of GC-treated mice, or control-treated tumor-bearing mice depleted or not of Tregs (D) (n=5 per group), and peripheral blood analysis of CD4$^+$ Foxp3$^+$ cells (E). Arrow indicates start of treatment (D).

Data are expressed as mean ± SEM; unpaired t-test (A, B), simple linear regression (C), two-way ANOVA (D) or one-way ANOVA (E). *, $P < 0.05$; **, $P < 0.01$; ***, $P < 0.001$; ****, $P < 0.0001$; ns, not significant.
