# Supplementary material for: Glucocorticoids unleash immune-dependent melanoma control through inhibition of the GARP/TGF-β axis
Source: Cancer Discov. Author manuscript; Available in PMC 2025 Oct 23. (PMC7618275; doi:10.1158/2159-8290.CD-24-1224)
Supplement: 12 [file EMS209516-supplement-12.pdf]

Figure S6

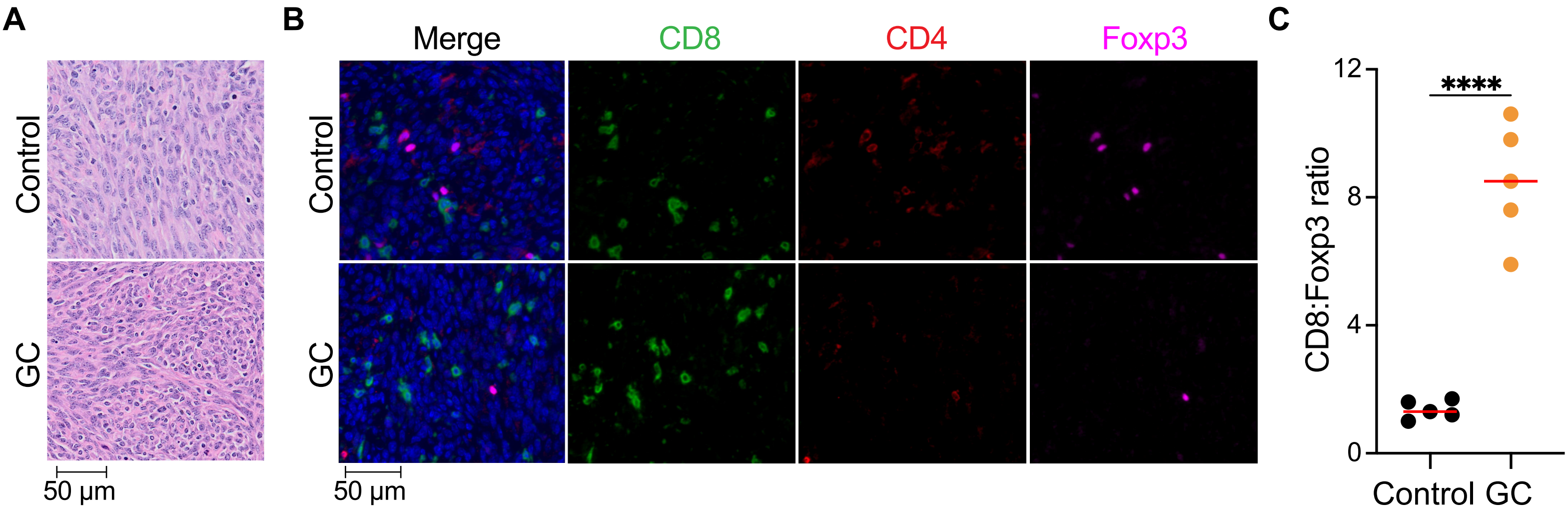
A
Control
GC
50 µm
B
Merge
CD8
CD4
Foxp3
Control
GC
50 µm
C
12
8
4
0
CD8:Foxp3 ratio
****
Control
GC

Supplementary Figure 6. GC treatment spares intratumoral $CD8^+$ T cells, resulting in an increased ratio of $CD8^+$ T cells to Tregs.

(**A**) High magnification Hematoxylin & Eosin images of day 5 post control or GC-treated tumors.

(**B**) Representative immunofluorescence images of control and GC-treated melanomas showing individual stains used (n=5 per group).

(**C**) CD8:Foxp3 ratio from immunofluorescence analysis of control and GC-treated melanomas (n=5 per group).

Data are expressed as mean; unpaired t-test (C). ****, $P < 0.0001$.
