# Supplementary material for: Glucocorticoids unleash immune-dependent melanoma control through inhibition of the GARP/TGF-β axis
Source: Cancer Discov. Author manuscript; Available in PMC 2025 Oct 23. (PMC7618275; doi:10.1158/2159-8290.CD-24-1224)
Supplement: 13 [file EMS209516-supplement-13.pdf]

Figure S7

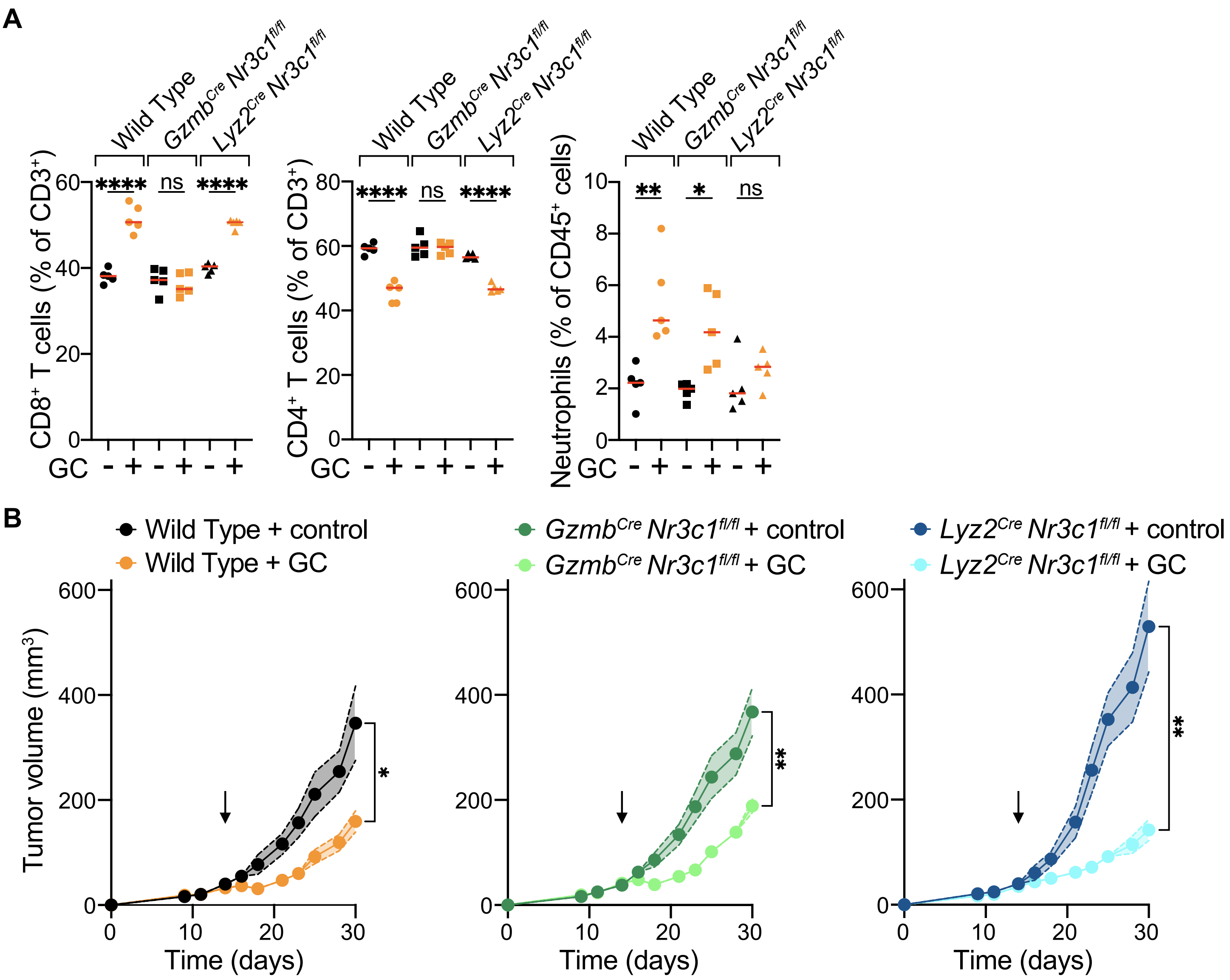
A
Wild Type
GzmbCre Nr3c1fl/fl
Lyz2Cre Nr3c1fl/fl
CD8+ T cells (% of CD3+)
CD4+ T cells (% of CD3+)
Neutrophils (% of CD45+ cells)
****
ns
**
*
GC
- + - + - +
B
Wild Type + control
Wild Type + GC
GzmbCre Nr3c1fl/fl + control
GzmbCre Nr3c1fl/fl + GC
Lyz2Cre Nr3c1fl/fl + control
Lyz2Cre Nr3c1fl/fl + GC
Tumor volume (mm3)
Time (days)

Supplementary Figure 7. GCs do not act on immune cells to trigger tumor control.

(**A**) Peripheral blood $CD8^+$ T (left panel) and $CD4^+$ T (middle panel) cells (as a percentage of $CD3^+$ T cells), and neutrophils (right panel; as a percentage of total leukocytes) after 2 weeks of control or GC treatment in wild type, $\textit{Gzmb}^{Cre}$ $\textit{Nr3c1}^{fl/fl}$ and $\textit{Lyz2}^{Cre}$ $\textit{Nr3c1}^{fl/fl}$ mice (n=5 per group).

(**B**) Growth profiles of melanomas implanted in wild type (left), $\textit{Gzmb}^{Cre}$ $\textit{Nr3c1}^{fl/fl}$ (middle) and $\textit{Lyz2}^{Cre}$ $\textit{Nr3c1}^{fl/fl}$ (right) mice following control or GC treatment (n=5 per group).

Data are expressed as mean ± SEM; one-way ANOVA (A) or two-way ANOVA (B). *, $P < 0.05$; **, $P < 0.01$; ****, $P < 0.0001$; ns, not significant. Arrow indicates start of treatment.
