# Supplementary material for: Glucocorticoids unleash immune-dependent melanoma control through inhibition of the GARP/TGF-β axis
Source: Cancer Discov. Author manuscript; Available in PMC 2025 Oct 23. (PMC7618275; doi:10.1158/2159-8290.CD-24-1224)
Supplement: 14 [file EMS209516-supplement-14.pdf]

Figure S8

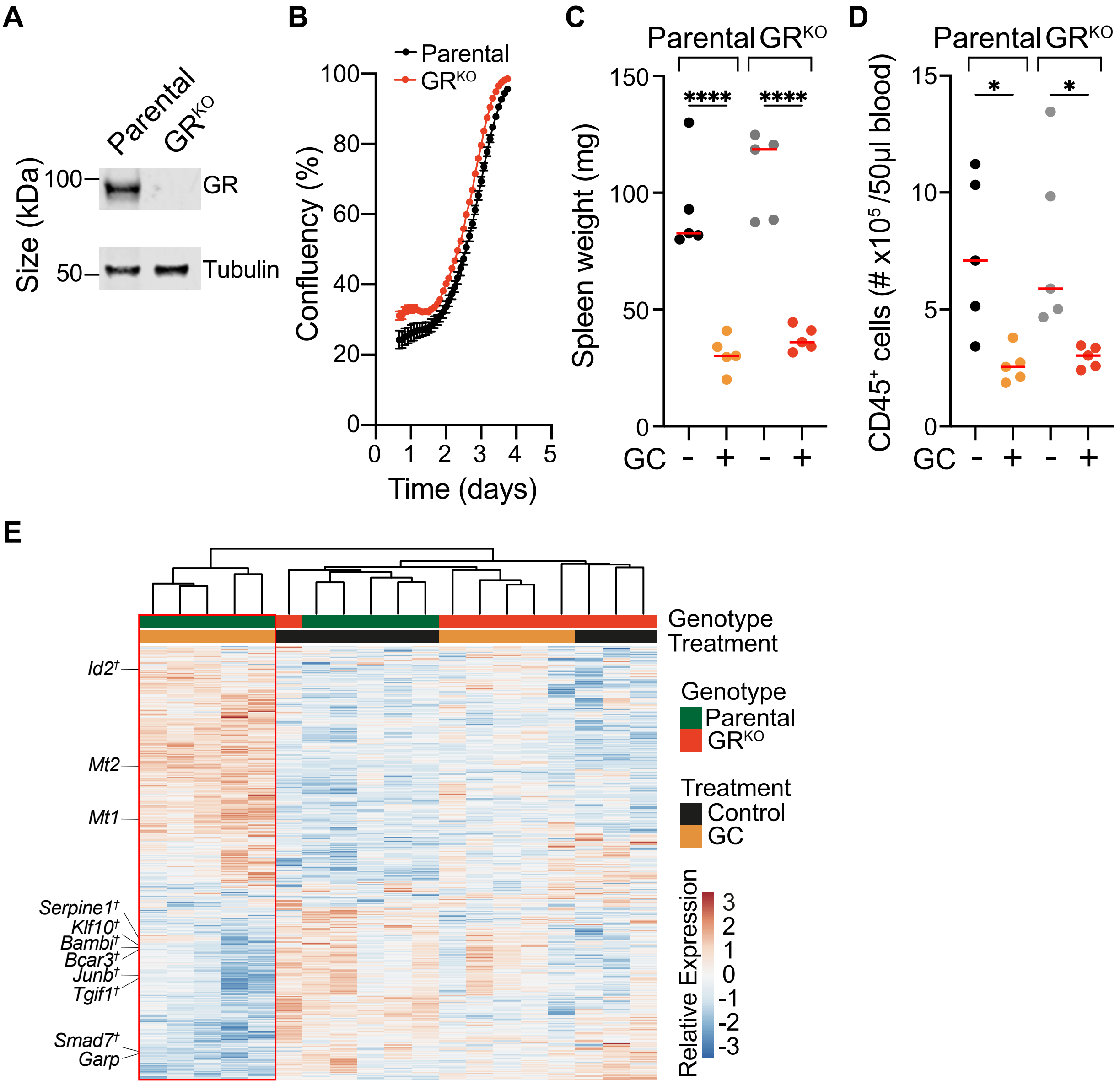

A
Parental
GRKO
Size (kDa)
100
50
GR
Tubulin
B
Parental
GRKO
Confluency (%)
Time (days)
C
Parental GRKO
Spleen weight (mg)
****
GC - + - +
D
Parental GRKO
CD45+ cells (# x10^5 /50µl blood)
*
GC - + - +
E
Genotype
Treatment
Parental
GRKO
Control
GC
Relative Expression
Id2†
Mt2
Mt1
Serpine1†
Klf10†
Bambi†
Bcar3†
Junb†
Tgif1†
Smad7†
Garp

Supplementary Figure 8. Topical GC treatment induces comparable systemic leukocyte reduction in parental and GR^{KO} tumor-bearing mice.

(**A**) Western blot of GR in parental and GR^{KO} 20967 melanoma cells.

(**B**) *In vitro* growth of parental and GR^{KO} 20967 melanoma cells.

(**C**) Spleen weight 5 days post treatment start with control or GC (n=5 per group).

(**D**) Peripheral blood $CD45^+$ cell count at day 14 on GC or control treatment (n=5 per group).

(**E**) Unsupervised clustering of differentially expressed genes (DEGs) in parental or GR^{KO} tumors with or without GC treatment following RNA sequencing *in vivo* (n=4-5 mice per group). *Mt1* and *Mt2* (GC-inducible), *Garp*, and members of Hallmark TGF-β signaling gene set[†] indicated.

Data are expressed as mean ± SEM; one-way ANOVA (C,D). *, $P < 0.05$; ****, $P < 0.0001$; ns, not significant.
