# Supplementary material for: Glucocorticoids unleash immune-dependent melanoma control through inhibition of the GARP/TGF-β axis
Source: Cancer Discov. Author manuscript; Available in PMC 2025 Oct 23. (PMC7618275; doi:10.1158/2159-8290.CD-24-1224)
Supplement: 15 [file EMS209516-supplement-15.pdf]

Figure S9

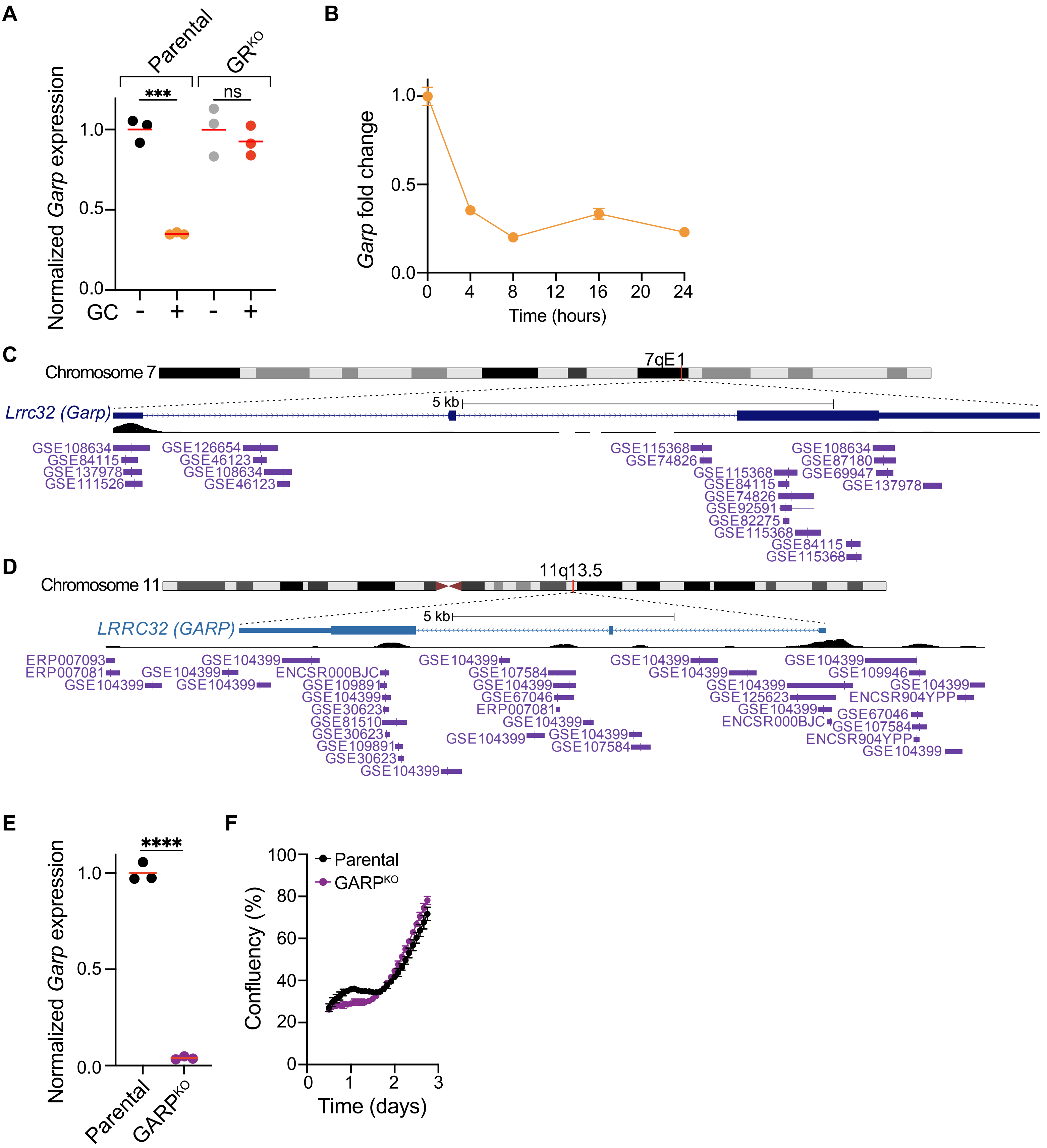

A
Parental
GR^KO
***
ns
Normalized Garp expression
1.0
0.5
0.0
GC - + - +
B
Garp fold change
Time (hours)
C
Chromosome 7
7qE1
Lrrc32 (Garp)
5 kb
D
Chromosome 11
11q13.5
LRRC32 (GARP)
5 kb
E
****
Normalized Garp expression
Parental
GARP^KO
F
Parental
GARP^KO
Confluency (%)
Time (days)

Supplementary Figure 9. GARP is a direct transcriptional target of GCs.

(**A**) GARP mRNA expression by qPCR of parental and GR^{KO} melanoma cells 24-hours post GC treatment *in vitro*. Data are expressed as normalized to *Hprt* and the untreated cell population.

(**B**) Time course analysis of GARP mRNA expression by qPCR over 24 hours in GC-treated parental 20967 melanoma cells. Data are expressed as normalized to *Hprt* and baseline GARP levels.

(**C, D**) Ch-IP sequencing data showing binding sites of GR (purple) in GARP (Lrrc32) gene in mice (C) and humans (D) in indicated published studies (purple labels).

(**E**) GARP expression in parental and $GARP^{KO}$ cells by PCR.

(**F**) *In vitro* growth of parental and $GARP^{KO}$ 20967 melanoma cells.

Data are expressed as mean ± SEM; one-way ANOVA (A) and unpaired t-test (E). ***, $P < 0.001$; ****, $P < 0.0001$; ns, not significant.
