# Supplementary material for: Glucocorticoids unleash immune-dependent melanoma control through inhibition of the GARP/TGF-β axis
Source: Cancer Discov. Author manuscript; Available in PMC 2025 Oct 23. (PMC7618275; doi:10.1158/2159-8290.CD-24-1224)
Supplement: 16 [file EMS209516-supplement-16.pdf]

Figure S10

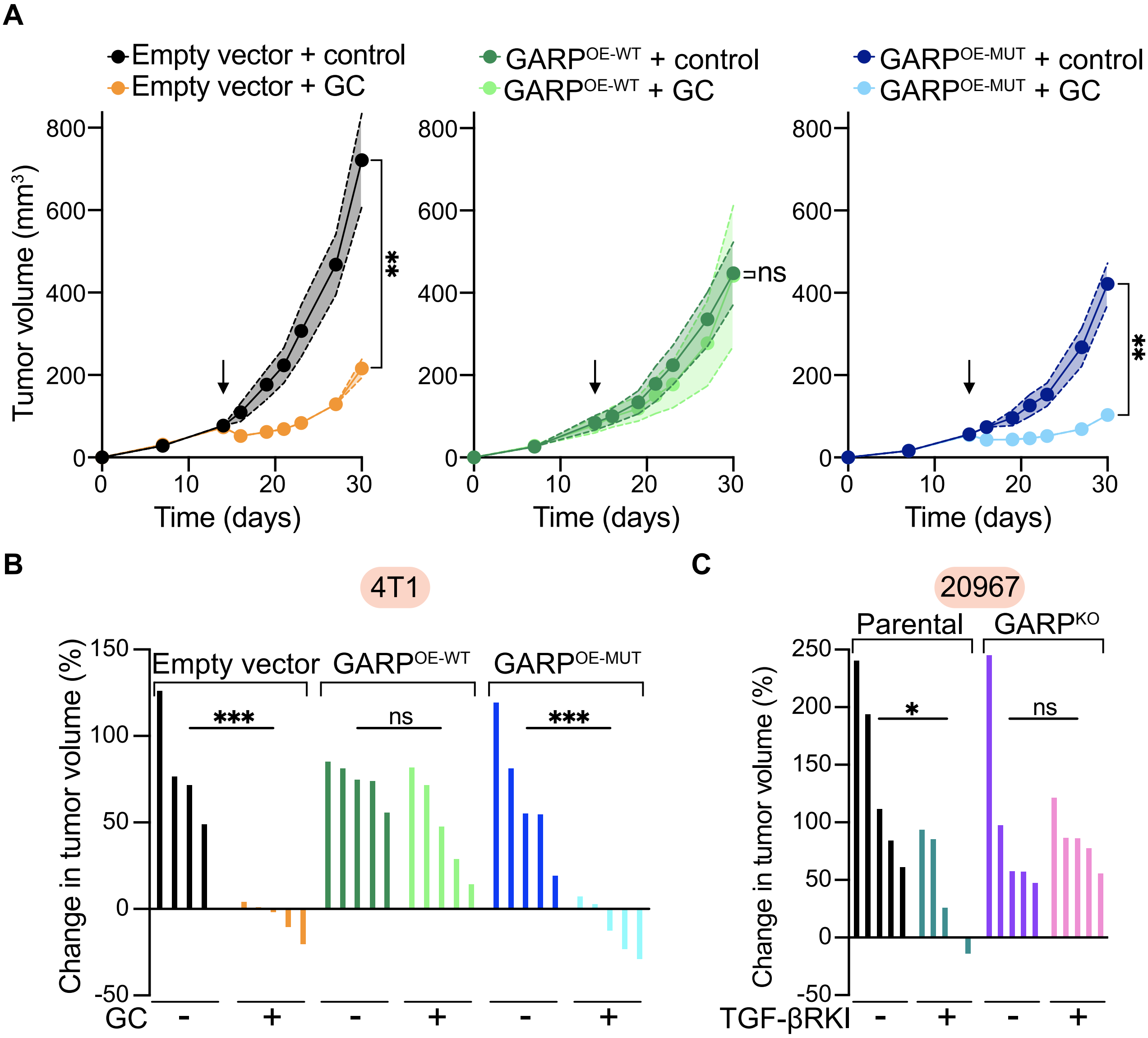
A
Empty vector + control
Empty vector + GC
GARP^{OE-WT} + control
GARP^{OE-WT} + GC
GARP^{OE-MUT} + control
GARP^{OE-MUT} + GC
Tumor volume (mm^3)
Time (days)
**
ns
**
B
4T1
Change in tumor volume (%)
Empty vector
GARP^{OE-WT}
GARP^{OE-MUT}
***
ns
***
GC
-
+
C
20967
Parental
GARP^{KO}
*
ns
TGF-βRKI

Supplementary Figure 10. The TGF-β activating function of GARP is required for GC-induced tumor control.

(**A**) Growth profiles of empty vector (left), $GARP^{OE-WT}$ (middle) and $GARP^{OE-MUT}$ (right) melanomas following control or GC treatment (n=5 per group). Arrow represents start of treatment.

(**B**) Waterfall plots showing percentage change in tumor volume on day 5 of treatment of control and GC-treated empty vector, $GARP^{OE-WT}$ and $GARP^{OE-MUT}$ 4T1 breast cancer tumors (n=5 per group).

(**C**) Waterfall plots showing percentage change in tumor volume on day 5 of treatment of control and TGF-βRKI-treated parental or $GARP^{KO}$ melanomas (n=5 per group). Data are expressed as mean ± SEM; two-way ANOVA (A) and one-way ANOVA (B, C). *, P<0.05; ***, P < 0.001; ns, not significant.
