# Supplementary material for: Glucocorticoids unleash immune-dependent melanoma control through inhibition of the GARP/TGF-β axis
Source: Cancer Discov. Author manuscript; Available in PMC 2025 Oct 23. (PMC7618275; doi:10.1158/2159-8290.CD-24-1224)
Supplement: 17 [file EMS209516-supplement-17.pdf]

Figure S11

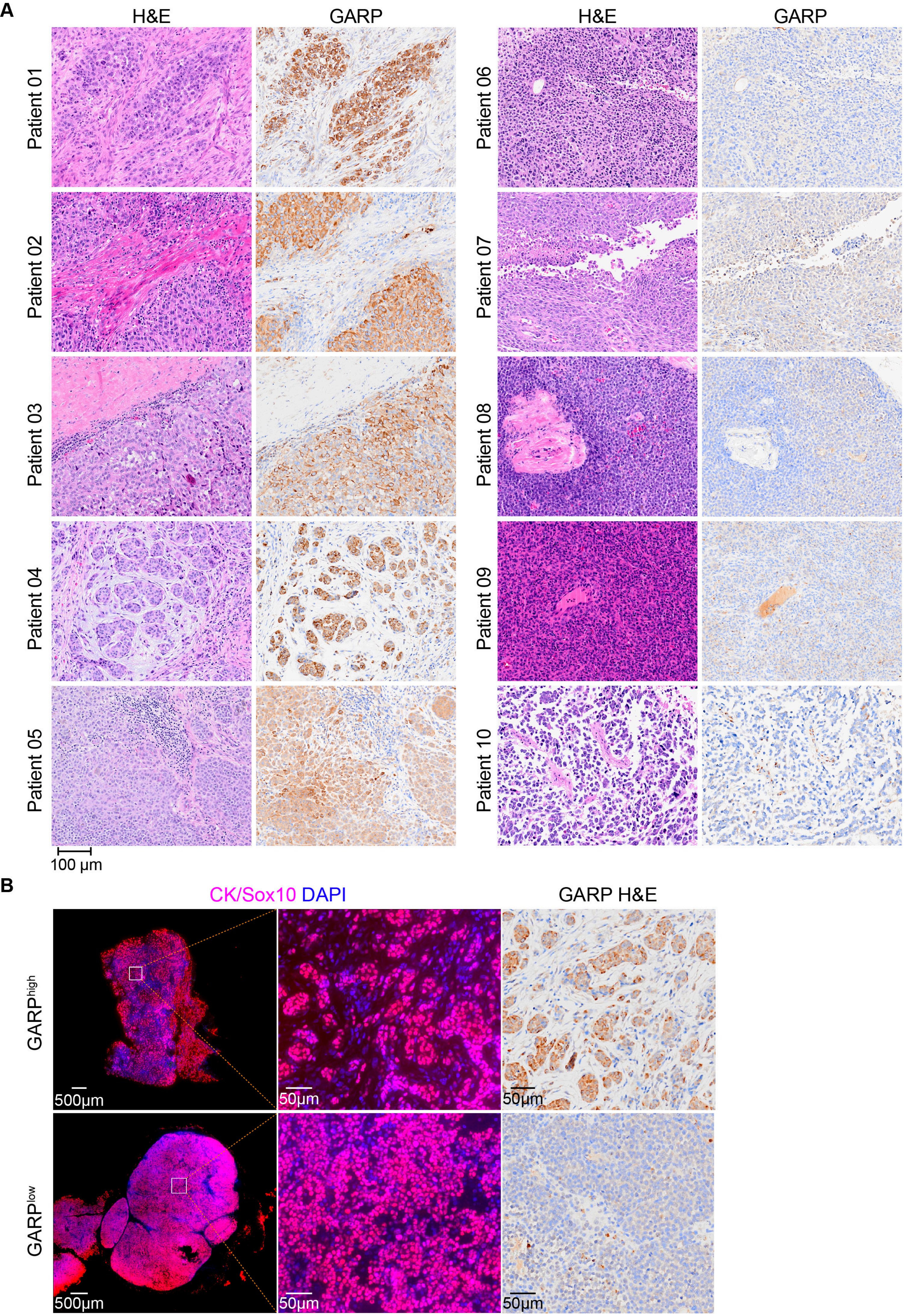

A
H&E
GARP
H&E
GARP
Patient 01
Patient 02
Patient 03
Patient 04
Patient 05
Patient 06
Patient 07
Patient 08
Patient 09
Patient 10
100 µm
B
CK/Sox10 DAPI
GARP H&E
GARPhigh
GARPlow
500µm
50µm
50µm
500µm
50µm
50µm

Supplementary Figure 11. Cancer cell-intrinsic GARP expression is markedly heterogeneous across melanoma patients.

(**A**) Representative immunohistochemistry images of 10 melanoma patients (patients 1-5 demonstrating high GARP levels; patients 6-10 demonstrating low GARP levels; Patients 1&2 and 5&6 shown in Fig. 7A).

(**B**) Staining of CK/Sox10, DAPI and GARP on serial sections of patient melanoma biopsies.
