# Supplementary material for: Glucocorticoids unleash immune-dependent melanoma control through inhibition of the GARP/TGF-β axis
Source: Cancer Discov. Author manuscript; Available in PMC 2025 Oct 23. (PMC7618275; doi:10.1158/2159-8290.CD-24-1224)
Supplement: 18 [file EMS209516-supplement-18.pdf]

## Figure S12

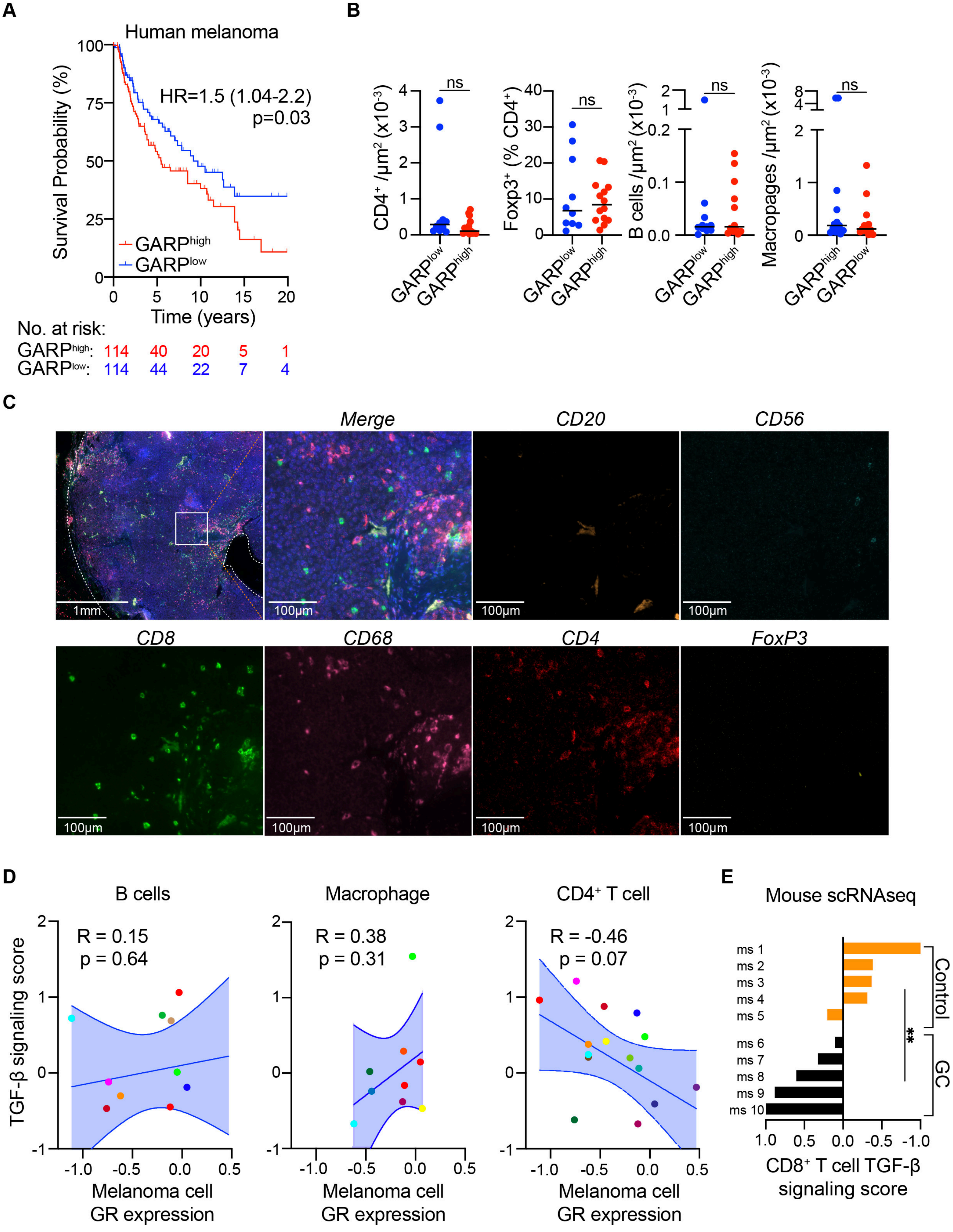
A
Human melanoma
HR=1.5 (1.04-2.2)
p=0.03
Survival Probability (%)
GARPhigh
GARPlow
Time (years)
No. at risk:
GARPhigh: 114 40 20 5 1
GARPlow: 114 44 22 7 4
B
CD4+ /µm2 (x10-3)
Foxp3+ (% CD4+)
B cells /µm2 (x10-3)
Macropages /µm2 (x10-3)
ns
GARPlow
GARPhigh
C
Merge
CD20
CD56
CD8
CD68
CD4
FoxP3
1mm
100µm
D
B cells
R = 0.15
p = 0.64
Macrophage
R = 0.38
p = 0.31
CD4+ T cell
R = -0.46
p = 0.07
TGF-β signaling score
Melanoma cell GR expression
E
Mouse scRNAseq
ms 1
ms 2
ms 3
ms 4
ms 5
ms 6
ms 7
ms 8
ms 9
ms 10
Control
GC
**
CD8+ T cell TGF-β signaling score

**Supplementary Figure 12. GARP$^{high}$ tumors have poorer prognosis but broadly comparable immune infiltrate composition.**

(**A**) Kaplan-Meier survival plot of TCGA melanoma patients (n=458) stratified by upper and lower quartile of GARP expression.

(**B**) Analysis of in-house melanoma samples from Figure 6B by multiparametric immunofluorescence. Prevalence of indicated populations shown.

(**C**) Representative images from human multiplex immunofluorescence analysis of in-house melanoma cohort (Figure 6B).

(**D**) Correlation of GR expression on melanoma cells with TGF-β signaling score on tumor-infiltrating B cells, macrophages and $CD4^+$ T cells from Jerby-Arnon et al[44].

(**E**) Mean hallmark "TGF-β Signaling" in tumor-infiltrating $CD8^+$ T cells from scRNA-sequencing analysis of control and GC-treated 20967 tumors (see Figure 3C). ms; mouse.

Hazard ratio (95% confidence interval), log-rank (Mantel-Cox) test (A), unpaired t-test (B, E) or linear regression with Pearson correlation (D). **, $P < 0.01$; ns; non-significant.
